# Poxviruses in Bats … so What?

**DOI:** 10.3390/v6041564

**Published:** 2014-04-03

**Authors:** Kate S. Baker, Pablo R. Murcia

**Affiliations:** 1 Wellcome Trust Sanger Institute, Hinxton, CB10 1SA, UK; 2 University of Glasgow Centre for Virus Research, Institute of Infection, Inflammation and Immunity, College of Medical, Veterinary and Life Sciences, University of Glasgow, Glasgow, G61 1QH, UK

**Keywords:** bats, poxviruses, host-range, emergence

## Abstract

Poxviruses are important pathogens of man and numerous domestic and wild animal species. Cross species (including zoonotic) poxvirus infections can have drastic consequences for the recipient host. Bats are a diverse order of mammals known to carry lethal viral zoonoses such as Rabies, Hendra, Nipah, and SARS. Consequent targeted research is revealing bats to be infected with a rich diversity of novel viruses. Poxviruses were recently identified in bats and the settings in which they were found were dramatically different. Here, we review the natural history of poxviruses in bats and highlight the relationship of the viruses to each other and their context in the *Poxviridae* family. In addition to considering the zoonotic potential of these viruses, we reflect on the broader implications of these findings. Specifically, the potential to explore and exploit this newfound relationship to study coevolution and cross species transmission together with fundamental aspects of poxvirus host tropism as well as bat virology and immunology.

## 1. Background and Significance

Poxviruses are double-stranded DNA viruses with large genomes (up to 300 kb) belonging to the family *Poxviridae*. The family is divided into the invertebrate-infecting *entomopoxvirinae* and chordate-infecting *chordopoxvirinae*. The latter subfamily is further divided into ten genera and contains many important infectious agents of both animals and humans. The now-eradicated Variola virus (VARV, the causative agent of smallpox) illustrates the potential consequences of poxvirus infections having arguably caused more deaths in human history than any other infectious agent [1]. Aside from humans, chordopoxviruses are also found in a multitude of terrestrial, aquatic and arboreal animal species from diverse taxa e.g., crocodiles, sea lions, birds, camels, *etc.* [2,3] and many poxviruses are capable of infecting multiple host species and cause cross-species (including zoonotic) infections [4]. For example, monkeypox virus has been recognized as a zoonotic agent since the 1970s and is classed a bioterrorism agent [5]. Further to human disease burdens, cross species infections of poxviruses between non-human species can also have devastating consequences e.g., the near-extinction of red squirrels in the UK after the introduction of squirrelpox with grey squirrels from the USA [6]. Owing to the significance of these zoonotic and cross-species poxvirus infections, poxvirus host range is a key area of research.

Poxviruses exhibit a heterogeneous host range with some poxviruses having a very broad host range (e.g., cowpox infects rodents, dogs, cats, horses, cows, primates including humans), and others being very specific (e.g., VARV is a human only pathogen). Although some poxvirus genera are known to exhibit broad host tropisms (e.g., orthopoxviruses) and are consequently thought to manifest greater zoonotic risks [7], phylogenetic relatedness among viruses is not indicative of poxvirus host range [8]. In fact, determinants of poxvirus host range are poorly understood and viral tropism is not typically restricted at the level of cellular entry. Due to highly conserved virion proteins, most poxviruses can enter a wide variety of host cell types, with restriction of infection occurring downstream of entry (either through a lack of host factors or through the innate immune system) [1,9,10]. Consequently, changes in poxvirus host range are typically determined by changes in virus genome complement (e.g., gene duplication/gain/loss) that allow for subversion of host restriction rather than point mutations [8,11,12], as is the case for some viruses e.g. parvovirus and influenza [13,14]. Genes that are known to cause shifts in poxvirus host range generally have functions relating to the interplay of the host innate immune mechanisms with the virus [8]. These genes are termed poxvirus host range genes and although approximately 15 have already been identified [10], more work is needed to fully understand their restriction mechanisms and to identify novel determinants of poxvirus host range.

**Figure 1:**
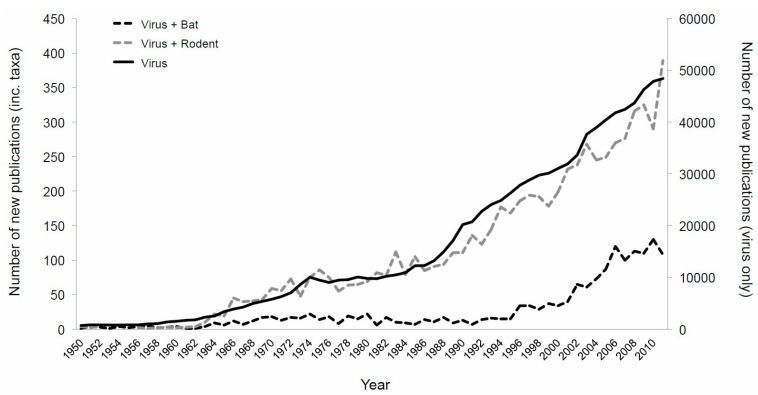
Number of publications recovered from SCOPUS by year when using the search term “virus” with (dashed lines, primary axis) and without (solid line, secondary axis) taxonomic orders.

Bats are an ancient, highly diverse order of mammals that are known to be reservoirs for a large number of viruses [15]. “Bats” is the collective term for some approximately 1200 species of mammals thought to have diverged some 50 million years ago (mya; comparatively humans and great apes are thought to have diverged ~5 mya) [16,17]. Second only in diversity to rodents, bats are subdivided into two suborders, commonly called megabats and microbats, on the basis of behavioral and physiological traits as well as molecular evidence [18]. There has been a recent increase in interest regarding the relationship of bats with viruses (Figure 1) as some species of bats are reservoir hosts for lethal viral zoonoses such as SARS coronaviruses [19,20], paramyxoviruses (e.g., Nipah and Hendra viruses) [21,22], and filoviruses (e.g., Ebola and Marburg virus) [23,24] and numerous lyssaviruses [25]. Outbreaks of disease attributable to bat-related zoonoses have high economic and human costs and their discovery has resulted in concerted research effort to isolate and characterize viruses from bat populations. Consequently, large numbers of previously unknown viruses have since been identified in bat populations for which the zoonotic potential is unknown, including novel influenza types and hepadnaviruses [26,27]. As a result, there has been well-grounded speculation that owing perhaps to physiological, ecological, evolutionary, and/or immunological reasons, bats may have a “special” relationship with viruses [15,28,29] and be particularly good viral reservoirs with exaggerated viral richness [30]. Indeed, a recent intensive study found that a single bat species likely carries ≥58 different viral species from only nine viral families [31]. As well as the obvious first step of considering the zoonotic potential of newly identified bat viruses, further exploring the impacts of these findings and the opportunities they present for multiple research fields is necessary to capitalize on these discoveries.

Poxvirus infections have recently been identified in bats, comprising part of the increase in viral families newly identified in this taxonomic order. Here, we review the current evidence of poxvirus infections in bats, present the phylogenetic context of the viruses within the *Poxviridae*, and consider their zoonotic potential. Finally, we speculate on the possible consequences and potential research avenues opened following this marrying of a pathogen of great historical and contemporary importance with an ancient host that has an apparently peculiar relationship with viruses; a fascinating and likely fruitful meeting whose study will be facilitated by recent technological advances and a heightened interest in bat virology.

## 2. The Natural History of Poxvirus Infection in Bats

There are three documented detections of poxviruses in bat populations under distinct circumstances (summarized in Table 1). The viruses were detected in animals from both bat suborders on three different continents. They had varied clinical impacts on their hosts and were phylogenetically dissimilar.

**Table 1:** Summary of poxvirus detections in bat species.

Bat Species	Bat Family	Geographical Site	Clinical Signs	Evidence	Genetic Characterization	Virus Name	Reference
*Eidolon helvum*	*Pteropodidae* (Megabat)	West Africa	Apparently healthy	Sequence detection	Partial sequencing (12kb)	*Eidolon helvum poxvirus 1*	[32]
*Eptesicus fuscus*	*Vespertilionidae* (Microbat)	USA	Tenosynovitis and osteoarthritis	EM ^A^ Isolated	Partial sequencing (19.5 kb)	*Eptesipoxvirus*	[35]
*Miniopterus schreibersii*	*Vespertilionidae* (Microbat)	Australia	Epidermal nodule	EM	NA ^B^	NA	[38]

### 2.1. Molecular Detection through Metagenomics

**Figure 2:**
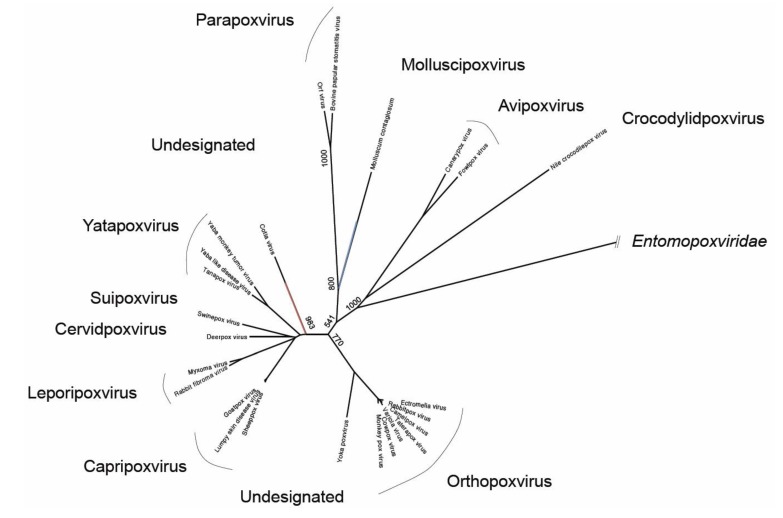
Neighbor-joining phylogenetic tree based on a 799aa alignment of the RAP94 protein of *Poxviridae* (please see Table S2 in Supplementary files). The approximate phylogenetic locations of *Eptesipox virus* (red) and *Eidolon helvum poxvirus 1* (blue) are shown. Bootstrap support (of 1000) of relevant nodes are shown.

Genetic sequence of one bat poxvirus was detected at high prevalence during active surveillance on apparently-healthy African straw-colored fruit bats (*Eidolon helvum*) [32]. Metagenomic analysis of pooled throat swabs collected from *E. helvum* in Ghana in 2009 contained poxvirus sequences most closely related with *Molluscum contagiosum* (MOCV) a human-only pathogen (Figure 2). Detected sequences were distributed across the MOCV genome and reconstructed sequences relating to 23 viral genes were deposited in GenBank as being derived from *Eidolon helvum poxvirus 1* [32]*.* Retrospective analysis of throat swabs from individual bats revealed a high prevalence of this *virus* in the apparently healthy study population with 13% (*n* = 5/40) of swabs containing poxviral DNA.

Notably, the detection of true poxvirus sequences in this metagenomic study, in which sequences related to multiple genes distributed throughout the genome were found and reconfirmed in individual throat swab samples, is distinct from the detection of poxvirus-like sequences described in other metagenomic studies performed on pooled bat feces, whose presence was ultimately attributed to the presence of other (non-pox) viruses or viral elements integrated into host genomes [33,34].

### 2.2. Viral Isolation and Clinical Infections

Between 2009 and 2011, a poxvirus associated with pathology (tenosynovitis and osteoarthritis) was detected in six adult big brown bats (*Eptescicus fuscus*, a microbat) sampled at a wildlife center in the North Western United States [35]. The clinical illness of the bats was progressive and ultimately led to their euthanasia. Histopathological examination of the joint lesions was indicative of poxvirus infection, which was confirmed by electron microscopy. The virus was successfully isolated on an African Green Monkey cell line (BSC40) and the genome was partially characterized (seven full protein coding sequences). Phylogenetic analysis revealed that the novel *Eptesipox virus* was most closely related with *Cotia virus*, a virus detected in sentinel suckling mice in Sao Paulo, Brazil in 1961 (Figure 2) [36,37].

Finally, a bat poxvirus was again detected in a clinical setting, in South Australia in 2009. The virus was identified as an incidental infection during investigation of an outbreak of parasitic skin disease in a population of Southern bentwing bats (*Miniopterus schreibersii bassanii*, a critically-endangered microbat species) [38]. Bats presented with white nodular skin lesions that contained encysted nematodes. However, in one of the twenty-one bats examined, an independent (non-nematode associated) lesion contained intracytoplasmic inclusion bodies indicative of poxvirus infection, which was confirmed with electron microscopy [38]. No further confirmation or characterization of the virus was reported, and both the epidemiology and consequent conservation implications of poxviral disease for this species remain unknown.

### 2.3. Interrelationships of Bat Poxviruses

The three detections of poxviruses in bat populations are distinct and inherently incomplete stories with very few common threads; high-prevalence detection in throat swabs from apparently healthy African megabats, severe joint disease in several North American microbats and, negligible though comorbid skin disease in an endangered Australasian microbat. Further to their varied clinical impact, the partial genetic characterization of the former two viruses shows that these viruses are genetically diverse. The two viruses are most closely related with the very distinct poxviruses, *Molluscum contagiosum virus* and *Cotia virus* respectively (Figure 2), and although only partially genetically characterized, a small (100 amino acids) region of overlap in their RAP94 proteins has only 62% amino acid identity (please see Table S1 in the Supplementary files). That this is as far as these new viruses can be contrasted demonstrates the dearth of information currently available for further investigation of poxviruses in bats.

## 3. The Zoonotic Potential of Bat Poxviruses

The finding of poxviruses in bats is not unique among wildlife taxa (in fact it would have been more surprising had they not been found to carry poxviruses) and there is no reason to believe they would have greater zoonotic potential than other animal poxviruses. Poxviruses with varying zoonotic potentials have been found in a broad range of wildlife taxa including hundreds of bird species, reptiles, marine mammals, macropods, marsupials, monotremes, ungulates, equids, and primates [1,2,5,39,40,41,42] and there is currently insufficient evidence available to determine what the zoonotic potential of bat poxviruses might be on this spectrum. For example, although *Eidolon helvum poxvirus 1* is closely related to MOCV, a human-only contagion, poxvirus-associated lesions mirroring MOCV-disease have also been found in horses, donkeys and a red kangaroo [41,42,43]. Similarly, the discovery of *Eptesipox virus* in North American brown bats is analogous to the discovery of the other North American poxviruses found in voles, skunks, raccoons and squirrels, which are also detected at high prevalence in their reservoir hosts [44,45]. Notably however, in the initial *Eptesipox virus* report, the authors comment that poxvirus infection manifesting as musculoskeletal disease (osteomyelitis) has also been reported in human VARV and Vaccinia virus (VACV) infections [35]. However, given that no bat poxviruses identified to date are orthopoxviruses, and the little information available, it is clear that much more detail is needed before the potential threat of bat poxviruses to man can be commented on. Notably however, the two hosts in which poxviruses have been identified are widely distributed across their respective continents (Africa and North America) and both habit urban areas, so have ample opportunities for contact with potential spillover hosts (i.e., humans and domestic animal species).

To determine the zoonotic risk posed by bat poxviruses there are, as for other novel viruses, a number of obvious and relatively straightforward investigations that can be done. Full genomic characterization of these viruses to identify known and putative poxvirus host range genes (discussed further below) would be an obvious step. Similarly, testing the *in vitro* host range of isolated viruses such as *Eptesipox virus* would help inform whether human and further animal cell lines are permissive for infection (*i.e*., that they contain the necessary host factors to support infection and do not contain antiviral components that restrict infection). Serological and clinical surveillance of human populations for poxvirus infections in geographical regions near detection sites, and/or overlapping with bat home ranges would be a direct approach that would provide samples useful for evaluating multiple candidate zoonoses. Whether bat poxviruses pose a zoonotic threat will likely comprise part of the future research agenda as these investigations are prudent for the discovery of all novel viruses. However, our current knowledge on bat poxviruses does not allow us to make firm predictions about their ability to infect humans.

## 4. Future Directions

Irrespective of their potential role as zoonotic agents however, the study of poxviruses in bats opens unique avenues of highly relevant research for multiple research fields beyond the individual host-pathogen relationships. Further field (*in situ*), *in vitro* and *in silico* studies could elucidate the possible coevolution, cross species infections and mechanisms of host range restriction of bat poxviruses, the implications of which are relevant for bat ecologists, virologists and emerging infectious disease specialists (including those with a specific interest in bats) alike.

### 4.1. Coevolution of Bats and Poxviruses

It is likely that comparative phylogenetics of bats and poxviruses would inform and deepen our understanding of origins and evolution of both elements. Bats and poxviruses are diverse host and pathogen taxa respectively and given their 0.5 million years of likely co-existence [46], there is surely a vast amount of knowledge to be gained by studying the phylogenetic relationships between bats and poxviruses. Further sampling of bat populations for poxviruses would undoubtedly dramatically expand the poxvirus phylogeny, as has occurred subsequent to the study of other viral taxa in bat populations [47,48,49,50,51,52,53]. Comparative phylogenetics of bats and their poxviruses could differentiate between ancient co-speciation, or a more recent introduction and dissemination, of poxviruses among bat species. The two thus far partially characterized bat poxviruses are quite distinct from each other and are both relatively basal (*i.e*., have older most recent common ancestors with other extant viruses) in the poxvirus phylogeny when compared with other mammalian-infecting poxviruses. It is possible that if evidence of coevolution between bats and poxviruses were present, as has been suggested for the North American poxviruses [44], this could inform the phylogenies of both bats and poxviruses which are complicated by convergent evolution and horizontal gene transfer respectively [54,55,56]. In addition to allowing the study of co-evolution, such studies provide the context for the identification of cross-species infections.

### 4.2. Cross Species Infections

With concerted research effort to identify reservoir species of bat poxviruses and cross species infections of poxviruses in bats could be identified and would have important implications for both bat and zoonotic-disease specialists. Continued serological and molecular studies of naturally infected bat populations would allow the clinical effect and ecological impact of cross species poxvirus infections in bats to be assessed. We already noted that poxvirus infections across species barriers can devastate wildlife populations (e.g., squirrelpox, see introduction), an effect so severe that it was used to control introduced rabbit species in Australia in the 1950s [57]. White nose syndrome, a fungal pathogen causing massive die offs in North American bat populations, is an unfortunate contemporary example of the severe ecological impacts that emerging pathogens can have on bat populations [58,59]. Hence, from an ecological perspective if a bat poxvirus, e.g., *Eptesipox virus* with its severe disease manifestations, were an emerging cross-species infection it would be useful to identify this rapidly, especially in already endangered species as is the case of the Southern bentwing bat in which a poxvirus was reported. Further to the conservation implications of such research, combining data regarding cross species infection and ecological aspects of host taxa (e.g., behavior, habitat, range overlap, host relatedness) will likely inform key concepts of virus sharing among bat species, as has been done with lyssaviruses [60,61].

### 4.3. Mechanisms of Poxvirus Host Tropism

Given the heightened interest in bat virology, further analysis of bat poxviral isolates from both within- and cross-species infections will allow for a deeper understanding of the extent and mechanisms of poxvirus host restriction. Many bat cell lines have now been developed [62,63,64,65,66], and at least one of these allows productive poxvirus infection [62]. Such tools will allow the *in vitro* refinement of host range definitions beyond detection in the field. Furthermore, full genome sequencing information of poxviruses (now a comparatively easy and cost effective task) would facilitate the *in silico* identification of poxvirus host range gene orthologues, as recently done by Bratke and colleagues who performed a systematic survey for the presence of known poxviral host range genes on among chordopoxviruses [3]. Furthermore, applying new bioinformatics tools to genomic sequence information and host range data could facilitate the identification of novel host-range determinants, perhaps even unique to bat poxviruses [12,67]. In addition, with the aforementioned *in vitro* tools in place, hypothetical host range genes can be validated, advancing our fundamental knowledge of poxvirus host range restriction.

### 4.4. Bat Immunology and Virology

Finally, and most speculatively, the identification of genes involved in poxvirus host range restriction in bats may represent a unique opportunity to study bat immunology, which may have broader implications for their confirmed roles as zoonotic reservoirs. Since genes that interplay with the host innate immune system, not those involved with cell entry, are typically responsible for host range determination in poxviruses [8,9], the identification of bat-unique poxvirus host range genes could facilitate the cognate identification of (possibly novel) host immune factors. This is particularly important for bats as they potentially have antiviral immunity distinct from our own, which seemingly allows them to harbor numerous human pathogens viruses asymptomatically [29]. Some preliminary evidence of this distinction existing for poxviruses is that in the single described report of infection of bat cell lines with poxviruses, bat cells were found to behave very differently from other mammalian cell lines, being susceptible to a highly attenuated strain of vaccinia virus [62]. With several bat genomes recently sequenced [68] and the capabilities of newer proteomic approaches, it is realistic that novel non-orthologous innate immune factors of bats (if they exist) could be identified. That these novel immune factors might then be candidate therapeutics against a range of viral zoonoses for which bats are the natural reservoir is an exciting, if not fantastical, point to ponder.

## 5. Concluding Remarks

Recent advances in the study of bats and their viruses as well as the current biotechnological revolution leave us in a position to explore questions of virology as never before. The recent detection of poxviruses in some bat species has occurred consequent to a heightened interest in bats’ role as viral reservoirs. These new findings enable us to ask many exciting and important questions about both bats and poxviruses independently as well as their ecological and evolutionary relationships. Integrating the new and exciting tools of the ‘omics revolution with traditional laboratory and field studies allow us to interrogate these questions as never before.

## Supplementary Files

Supplementary File 1 Supplementary Information (PDF, 496 KB)
