# Supplementary material for: Poxviruses in Bats … so What?
Source: Viruses. 2014 Apr 3;6(4):1564–77. doi: 10.3390/v6041564 (PMC4014710; doi:10.3390/v6041564)
Supplement: Supplementary File 1 — Supplementary Information (PDF, 496 KB) [file viruses-06-01564-s001.pdf]

Table S1. Pairwise gaps (upper right) and amino acid identity (lower left) over a 100 aa alignment of *Poxviridae* RAP94-orthologues.

**Genome**	**Reference**	**1**	**2**	**3**	**4**	**5**	**6**	**7**	**8**	**9**	**10**	**11**	**12**	**13**	**14**	**15**	**16**	**17**	**18**	**19**	**20**	**21**	**22**	**23**	**24**	**25**	**26**	**27**	**28**
Sheeppox virus	1		0	0	0	0	0	0	0	0	0	0	0	0	1	0	0	0	0	0	0	2	1	0	0	0	0	0	0
Lumpy skin disease	2	100		0	0	0	0	0	0	0	0	0	0	0	1	0	0	0	0	0	0	2	1	0	0	0	0	0	0
Goatpox virus	3	100	100		0	0	0	0	0	0	0	0	0	0	1	0	0	0	0	0	0	2	1	0	0	0	0	0	0
Deerpox virus	4	87	87	87		0	0	0	0	0	0	0	0	0	1	0	0	0	0	0	0	2	1	0	0	0	0	0	0
Swinepox virus	5	88	88	88	87		0	0	0	0	0	0	0	0	1	0	0	0	0	0	0	2	1	0	0	0	0	0	0
Myxoma virus	6	85	85	85	84	83		0	0	0	0	0	0	0	1	0	0	0	0	0	0	2	1	0	0	0	0	0	0
Rabbit fibroma virus	7	85	85	85	84	85	97		0	0	0	0	0	0	1	0	0	0	0	0	0	2	1	0	0	0	0	0	0
Yaba like disease virus	8	80	80	80	80	83	83	86		0	0	0	0	0	1	0	0	0	0	0	0	2	1	0	0	0	0	0	0
Tanapox virus	9	79	79	79	79	82	82	85	99		0	0	0	0	1	0	0	0	0	0	0	2	1	0	0	0	0	0	0
Yaba monkey tumor virus	10	77	77	77	80	82	81	83	94	93		0	0	0	1	0	0	0	0	0	0	2	1	0	0	0	0	0	0
Cotia virus	11	81	81	81	79	84	78	80	81	80	77		0	0	1	0	0	0	0	0	0	2	1	0	0	0	0	0	0
Cowpox virus	12	67	67	67	67	69	65	67	69	68	67	67		0	1	0	0	0	0	0	0	2	1	0	0	0	0	0	0
Rabbitpox virus	13	67	67	67	67	69	65	67	69	68	67	67	100		1	0	0	0	0	0	0	2	1	0	0	0	0	0	0
Ectromelia virus	14	66	66	66	66	68	64	66	68	67	66	66	98	98		1	1	1	1	1	1	3	2	1	1	1	1	1	1
Taterapox virus	15	67	67	67	67	69	65	67	69	68	67	67	100	100	98		0	0	0	0	0	2	1	0	0	0	0	0	0
Camelpox virus	16	67	67	67	67	69	65	67	69	68	67	67	100	100	98	100		0	0	0	0	2	1	0	0	0	0	0	0
Monkeypox virus	17	67	67	67	67	69	65	67	69	68	67	67	100	100	98	100	100		0	0	0	2	1	0	0	0	0	0	0
Variola virus	18	67	67	67	67	69	65	67	69	68	67	67	100	100	98	100	100	100		0	0	2	1	0	0	0	0	0	0
Yoka poxvirus	19	64	64	64	64	63	67	68	66	65	63	63	83	83	83	83	83	83	83		0	2	1	0	0	0	0	0	0
Molluscum contagiosum	20	67	67	67	67	70	68	68	66	65	65	67	71	71	70	71	71	71	71	68		2	1	0	0	0	0	0	0
Fowlpox virus	21	41	41	41	43	41	41	40	42	41	39	39	39	39	40	39	39	39	39	39	42		1	2	2	2	2	2	2
Canarypox virus	22	40	40	40	43	41	40	39	41	41	39	38	38	38	39	38	38	38	38	39	41	91		1	1	1	1	1	1
Bovine papular stomatitis virus	23	58	58	58	61	59	60	59	57	56	59	62	53	53	53	53	53	53	53	56	64	37	37		0	0	0	0	0
Orf virus	24	56	56	56	60	59	60	60	57	56	59	61	54	54	54	54	54	54	54	55	65	38	38	87		0	0	0	0
Nile crocodilepox virus	25	44	44	44	45	44	44	44	46	45	44	40	40	40	40	40	40	40	40	40	42	39	38	38	36		0	0	0
Eidolon helvum poxvirus_1_th6839062	26	65	65	65	69	67	68	67	63	62	62	67	68	68	68	68	68	68	68	65	82	40	39	65	64	43		0	0
Eptesipox virus	27	73	73	73	72	73	73	73	72	71	67	71	70	70	69	70	70	70	70	67	67	40	40	56	56	42	62		0
Amsacta moorei entomopoxvirus	28	9	9	9	8	8	9	9	9	10	7	8	5	5	5	5	5	5	5	6	4	5	4	6	5	6	5	9	
Melanoplus sanguinipes entomopoxvirus	29	8	8	8	8	8	9	10	8	7	6	7	3	3	3	3	3	3	3	5	3	7	7	3	3	7	4	8	34

Table S2. Genbank accession numbers of viruses used in phylogenetic tree.

Name	Genbank Accession
Amsacta moorei entomopoxvirus	NP_64836
Bovine papular stomatitis virus	NP_957969
Lumpy skin disease	NP_150509
Melanoplus sanguinipes entomopoxvirus	NP_48189
Nile crocodilepox virus	YP_784294
Rabbit fibroma virus	NP_51961
Yaba like disease virus	NP_73460
Yaba monkey tumor virus	NP_938330
Camelpox virus	AAG37572
Canarypox virus	NP_955210
Cotia virus	YP_5296279
Cowpox virus	ADZ30718
Deerpox virus	YP_227459
Ectromelia virus	NP_671604
Fowlpox virus	AF198100_132
Goatpox virus	YP_1293266
Molluscum contagiosum	NP_44036
Monkeypox virus	AGF36998
Myxoma virus	NP_51786
Orf virus	NP_957837
Rabbitpox virus	AAS49804
Sheeppox virus	NP_659647
Swinepox virus	NP_570232
Tanapox virus	YP_1497070
Taterapox virus	YP_717411
Variola virus	CAA47586
Yoka poxvirus	AEN03671
